# Cognitive Outcomes During COVID-19 Confinement Among Older People and Their Caregivers Using Technologies for Dementia: Protocol for an Observational Cohort Study

**DOI:** 10.2196/26431

**Published:** 2021-05-18

**Authors:** Jessica Marian Goodman-Casanova, Elena Dura-Perez, Gloria Guerrero-Pertiñez, Pilar Barnestein-Fonseca, Jose Guzman-Parra, Amanda Vega-Nuñez, Esperanza Varela-Moreno, Antonio Cuesta-Vargas, Fermin Mayoral-Cleries

**Affiliations:** 1 Department of Mental Health Regional University Hospital of Málaga Biomedical Research Institute of Malaga (IBIMA) Málaga Spain; 2 Faculty of Psychology University of Málaga Málaga Spain; 3 Department of Physiotherapy University of Málaga Biomedical Research Institute of Malaga (IBIMA) Málaga Spain

**Keywords:** caregiver, cognition, cognitive impairment, cohort, COVID-19, dementia, older people, informal caregivers, information and communications technologies, isolation, older adults, outcome, quality of life, social isolation, stress, technologies

## Abstract

**Background:**

The COVID-19 pandemic has led to worldwide implementation of unprecedented restrictions to control its rapid spread and mitigate its impact. The Spanish government has enforced social distancing, quarantine, and home confinement measures. Such restrictions on activities of daily life and separation from loved ones may lead to social isolation and loneliness with health-related consequences among community-dwelling older adults with mild cognitive impairment or mild dementia and their caregivers. Additionally, inadequate access to health care and social support services may aggravate chronic conditions. Home-based technological interventions have emerged for combating social isolation and loneliness, while simultaneously preventing the risk of virus exposure.

**Objective:**

The aim of this cohort study is to explore, analyze, and determine the impact of social isolation on (1) cognition, quality of life, mood, technophilia, and perceived stress among community-dwelling older adults with mild cognitive impairment or mild dementia and on the caregiver burden; (2) access to and utilization of health and social care services; and (3) cognitive, social, and entertainment-related uses of information and communication technologies.

**Methods:**

This study will be conducted in Málaga (Andalucía, Spain). In total 200 dyads, consisting of a person with mild cognitive impairment or mild dementia and his/her informal caregiver, will be contacted by telephone. Potential respondents will be participants of the following clinical trials: support, monitoring, and reminder technology for mild dementia (n=100) and television-based assistive integrated service to support European adults living with mild dementia or mild cognitive impairment (n=100).

**Results:**

As of May 2021, a total of 153 participants have been enrolled and assessed during COVID-19 confinement, of whom 67 have been assessed at 6 months of enrollment. Changes in the mean values of the variables will be analyzed relative to baseline findings of previous studies with those during and after confinement, using repeated-measures analysis of variance or the nonparametric Friedman test, as appropriate. The performance of multivariate analysis of covariance (ANCOVA) to introduce potential covariates will also be considered. Values of 95% CI will be used.

**Conclusions:**

If our hypothesis is accepted, these findings will demonstrate the negative impact of social isolation owing to COVID-19 confinement on cognition, quality of life, mood, and perceived stress among community-dwelling older adults with mild cognitive impairment and mild dementia, the impact on technophilia, caregiver burden, the access to and utilization of health and social care services, and the cognitive, social, and entertainment-related use of information and communication technologies during and after COVID-19 confinement.

**Trial Registration:**

ClinicalTrials.gov NCT04385797; https://clinicaltrials.gov/ct2/show/NCT04385797

**International Registered Report Identifier (IRRID):**

DERR1-10.2196/26431

## Introduction

The COVID-19 pandemic has forced government authorities worldwide to implement unprecedented restrictions to control its rapid spread and mitigate its impact [1]. In response to the outbreak, Spain declared, by royal decree (463/2020), a national emergency, with the exceptional measure of a nationwide lockdown enforcing social distancing, quarantining of those exposed to the virus, and home confinement of those who remain healthy, while allowing only essential outings [2]. This home-confinement by restricting movement to carry out activities of daily life and social distancing from loved ones may be a challenging and unpleasant experience for those who undergo it, leading them to experience social isolation and loneliness and having health-related consequences. Vulnerable populations at a higher risk are fragile community-dwelling older adults whose chronic conditions may be aggravated by the consequences of the confinement and, in particular, people with mild cognitive impairment or mild dementia (PMCI/MD) [3].

Social isolation (the absence of social contacts, interactions, or relationships with other individuals including family, friends, or neighbors) and loneliness (perception of isolation or feeling of being lonely) have been well established as risk factors for health-related consequences and quality of life (QoL) [4] (an individual's perception of their position in life in a cultural context in relation to their goals, expectations, standards, and concerns [5], including physical and mental health, social relationships, and participation in activities).

Numerous observational studies have reported associations between social isolation and an increased risk of dementia and cognitive decline in older adults [6-8]. Reduction of social contacts and lower levels of participation in social activities are associated with declines in global cognition, processing speed, executive function, and visuospatial abilities [9].

Facing novel and unknown situations is a potential stressor, especially when cognition may be compromised [10], and loneliness may help predict changes in depressive symptomatology [11]. A systematic review on social relationships and depression in later life, which included 37 studies (25 cross-sectional and 12 longitudinal studies), reported that having a decreased social network was significantly associated with depression in older adults [12].

Social isolation is likewise associated with a higher prevalence of other comorbid conditions including cardiovascular diseases [13], stroke [13], and the risk of premature mortality [14]. Furthermore, studies on social isolation and health-related behaviors have reported that older people who are isolated are more likely to have less health-related behaviors such as poor diets, tobacco use, heavy alcohol use, and a lack of physical activity [6,15].

The burden of COVID-19 exerts pressure on health care and social support services [2] and caregivers. Spain’s health care system is struggling to deliver emergency and intensive care. Routine interventions for service provision, such as primary and specialist care consultations, diagnostic testing, and nonemergency interventions, have been cancelled, postponed, or their delivery has shifted from an in-person to a technology-based format. Additionally, caregivers’ burden may be worsened in an attempt to reduce the risk of virus exposure among their care recipients.

In the information age, with the increased use of information and communication technologies (ICTs), home-based technological interventions [16] including smartphones, tablets, computers, smart televisions, virtual assistants, and ambient assistive devices have emerged for combating social isolation and loneliness. A challenge associated with these interventions is that vulnerable populations at a higher risk of becoming severely socially isolated owing to the COVID-19 pandemic, such as older people, may have negative attitudes and lack of enthusiasm toward the use of ICTs, thus having low technophilia [17]. Technophilia is described as the “attraction, enthusiasm of the human individual determined by the activities which involve the use of advanced technologies. It is expressed by easy adaptation to the social changes brought by technological innovations” [18].

International recommendations stress the need to urgently identify the needs of people with dementia and their caregivers and establish technological strategies for their assistance and support [19]. In response to the COVID-19 pandemic, studies have shown increased interest in technology among people with dementia [20] and have emphasized the challenges associated with the adoption of technology-based interventions by people with dementia [21], but few studies have explored the impact of technologies on social isolation in this population.

The aims of this study are threefold: (1) to explore the impact of social isolation on cognition, QoL, mood, technophilia, and perceived stress of community-dwelling older PMCI/MD and on caregiver burden; (2) to investigate how social isolation affects the access to and utilization of health care and social support services; and (3) to determine the health-related, cognitive, social, informative, and entertainment-related uses of ICTs during and after 6 months of COVID-19 confinement.

## Methods

### Study Design

This cohort study will be conducted in Málaga (Andalucía, Spain). The study will assess Cognitive Outcomes During COVID-19 confiNemeNt in Elderly and Their Caregivers Using Technologies for DEMentia (CONNECTDEM). Interviews will be conducted telephonically to guarantee the safest means to communicate during the COVID-19 pandemic. Researchers will contact participants by telephone, explain the study in detail, answer any questions which may arise, and ask those who are willing to participate in the study to provide consent. Interviews were conducted in May 2020. Follow-up assessments would be carried out after 6 months.

### Setting

Participants will be identified from the support, monitoring, and reminder technology for mild dementia (SMART4MD; trial# NCT03325699) [22] and television-based assistive integrated service to support European adults living with mild dementia or mild cognitive impairment (TV-AssistDem; trial# NCT03653234) [23] clinical trials, both of which aim to assess the effects of ICTs to support dementia by using a tablet-based health app and a television-based assistive integrated service, respectively. Participants will include people with self-perceived cognitive impairment or their caregiver’s perception of cognitive impairment that has been present for >6 months under primary and secondary care services, including those who are being followed-up at memory clinics, outpatient clinics, day hospitals, or other components of specialist mental health, geriatric medicine, and neurology services.

### Participants

The participant dyads will comprise PMCI/MD and their informal caregivers, defined as the person who provides support or care, spends the most time with the patient, is unwaged for this role, and does not participate in a formal network of organized care [24]. In total 200 dyads, 100 from the SMART4MD trial 100 from the TV-AssistDem trial, from both the intervention and control groups will be potential respondents. A dyad will be eligible for inclusion in this study only if they have participated in the SMART4MD or TV-AssistDem clinical trials and they consent to participating in this study.

### Outcome Measures

The primary outcome measure will be the change in cognition in PMCI/MDs. Additional assessments will be performed to assess secondary outcomes including the QoL, mood, technophilia, and perceived stress among PMCI/MDs; caregiver burden; access to and utilization of health and social care services; and cognitive, social, and entertainment-related use of ICTs.

Baseline assessments of cognition in PMCI/MDs prior to COVID-19 confinement (T0), their QoL, mood, and technophilia, and the QoL and burden of their caregivers will be compared with assessments carried out during COVID-19 confinement (T1) and at 6 months (T2). Additionally, perceived stress regarding the confinement situation, access to and utilization of health and social care services, and cognitive, social, and entertainment-related use of ICTs will be measured at T1 and T2. The assessments will be conducted under standardized conditions at the study center.

#### Primary Outcome: Cognition

The mini-mental state examination (MMSE) [25] will be performed to assess the cognitive function of the PMCI/MD. The most common cutoff scores for cognitive impairment and dementia range 23-27 out of 30. As telephonic interviews will be the safest means to communicate with the PMCI/MDs during and after the COVID-19 pandemic, a telephone-based cognitive assessment will be carried out using the 22-item telephonic version of the MMSE [26]. All items of the MMSE can be discussed through the telephone version, except for one question in the orientation section, regarding the floor on which the patient resides (as the researchers would not be able to ascertain that) and the last section assessing language and motor skills. In the telephonic version, we will ask the subject to repeat a phrase and name 1 item (for example: “Tell me, what is the name of the object you are using to talk to me?”). However, a second item will not be named, nor will the person be asked to follow a 3-stage command, read and carry out an instruction, write a sentence, or copy an intersecting pentagon as in the original version.

#### Secondary Outcomes

##### Patient QoL

The Quality of Life-Alzheimer’s Disease (QoL-AD) scale [27,28] is an instrument specifically designed to measure QoL in PMCI/MDs from the perspective of both the patient and the informal caregiver. It is a 13-item scale, which includes assessments of the person´s relationships with friends and family, financial situation, physical condition, mood, memory, and an overall assessment of life quality. Response are 4-point multiple-choice options (1=“poor,” 2=“fair,” 3=“good,” and 4=“excellent”). Scale scores range from 13 to 52, with higher scores indicating a greater QoL. As cognitive function may be compromised, informal caregivers will also complete the QoL-AD, in parallel with and on behalf of the PMCI/MD throughout the study.

The European QoL 5-dimension, 3-level instrument [29,30] is a standardized generic instrument consisting of a descriptive system and a visual analog scale. The descriptive system comprises five dimensions: mobility, self-care, usual activities, pain or discomfort, and anxiety or depression. Each dimension has three levels: no problems, moderate problems, and extreme problems. A 1-digit number expresses the level selected for that dimension. The digits can be combined into a 5-digit number that describes the patient's health state. The visual analog scale records the patient's self-rated health on a vertical scale, where the endpoints are “the best health you can imagine” and “the worst health you can imagine.” The European QoL 5-dimension, 3-level instrument has been shown to correlate well with the QoL-AD scale, indicating that using both measures in parallel is compatible [31].

##### Mood

The shortened form of the geriatric depression scale [32] will be used to assess mood. Of 15 items in this version, 10 indicate depression when answered positively, while the remaining 5 (items 1, 5, 7, 11, and 13) indicate depression when answered negatively. Scores of 0-4 are considered normal, 5-8 indicate mild depression, 9-11 indicate moderate depression, and 12-15 indicate severe depression.

##### Technophilia

The instrument for measuring older people’s attitudes toward technology [33] measures older people’s attitudes and enthusiasm toward health technology. This instrument refers to technophilia as a person’s enthusiasm for and positive feelings toward their technology use and the absence of fears and doubts some older people could have about their ability to manage using new technology. The 6 items of this instrument measure 2 factors related to technophilia: 3 items concerning technological enthusiasm and 3 items concerning technological anxiety. Responses are based on a 5-point Likert scale ranging from 1=“fully disagree” to 5=“fully agree.”

##### Perceived Stress

The perceived stress scale [34] measures the degree to which situations in one’s life are appraised as stressful. The scale comprises 10 questions regarding feelings and thoughts during the past month, which are rated on the basis of frequency (0=“never,” 1=“almost never,” 2=“sometimes,” 3=“fairly often,” and 4=“very often”). Scores are obtained by reversing the responses (eg, 0=4, 1=3, 2=2, 3=1, and 4=0) to the 4 positively stated items (items 4, 5, 7, and 8) and then summing the scores of all items.

##### Caregiver Burden

The 12-item Zarit burden interview [35,36] will be used to evaluate the informal caregivers’ burden. This scale has responses scored on a 5-point Likert scale (4=“nearly always,” 3=“quite frequently,” 2=“sometimes,” 1=“rarely,” and 0=“never”). It is a shortened version of the original scale and has been developed specifically for informal caregivers of PMCI/MDs and encompasses issues such as caregiver stress and the degree to which caregiving affects their health and social life. The total score ranges 0-48 (0-10=“no to mild burden,” 10-20=“mild to moderate burden,” and >20=“high burden”).

##### Service Utilization

The client service receipt inventory scale [37,38] will be used to evaluate the service utilization. This scale is an internationally used method for gathering data on service utilization and other domains relevant for economic analysis of mental health care. It has five sections: background client information, accommodation and living conditions, employment history, earnings and benefits, and a record of services usually used and information on informal caregiver support. The sections assessed will be consultations, admissions, and visits, grouped into subsections in accordance with hospital, specialist, primary, or home care. Treatment related to hospital admissions or illness exacerbation will also be assessed. The adaptability of this scale ensures that it is compatible with the study aims, context, participants’ likely circumstances, and the quantity and precision of information required.

##### Use of ICTs

Use of ICTs, including smartphones, tablets, computers, smart televisions, or other devices, to contact health care and social support services, stimulate cognition, facilitate social connectedness (through telephone calls, video calls, and text messages), to access information on COVID-19 and to enable entertainment.

##### Covariables

Covariables will involve sociodemographic data including marital status, level of education, and living arrangements, medical history, health perception management, changes in living arrangements due to the lockdown, presence of COVID-19 symptoms in PMCI/MDs or their relatives, the frequency of access to COVID-19–related information, and understanding of the information, ability to manage illnesses, provision of support for purchasing medication and food, coping stress tolerance, mental health, well-being, and self-perceived mood, sleep and rest, alterations in usual sleep patterns and the use of additional medication, and leisure activities including preferred physical, intellectual, recreational, and social activities. The collected variables and data collection time are shown in Table 1.

**Table 1:** Data collection table.

Measure	Data collection timepoint			
	Outcome variable	Baseline	During COVID-19 confinement	At 6 months
Informed consent	N/A^a^	✓^b^	✓	N/A
Sociodemographic data	N/A	✓	✓	✓
Mini-mental state examination	Primary	✓	✓	✓
Quality of Life-Alzheimer’s Disease scale	Secondary	✓	✓	✓
European quality of life 5-dimension, 3-level	Secondary	✓	✓	✓
Geriatric depression scale	Secondary	✓	✓	✓
Instrument for measuring older people’s attitudes toward technology	Secondary	X^c^	✓	✓
Perceived stress scale	Secondary	X	✓	✓
Client service receipt inventory	Secondary	✓	✓	✓
Zarit burden interview	Secondary	✓	✓	✓

^a^N/A: not applicable.

^b^Data were collected.

^c^Data were not collected.

### Statistical Analysis

The flow of participants will be shown schematically with counts and percentages in a CONSORT diagram. All variables collected will be summarized at baseline and at follow-up. Statistics considered for presentation for continuous measures in summary tables include the mean (SD), minima, and maxima, and if the criteria of normality are not met, the median and the first and third quartiles will be recorded. Categorical variables will be summarized as counts and percentages.

The change in means in the study variables will be analyzed in accordance with the results of previous trials (SMART4MD and TV-AssistDem) with those currently collected using repeated-measures analysis of variance or the nonparametric Friedman test as appropriate. The performance of multivariate analysis of covariance (ANCOVA) to introduce possible covariates will also be considered. A 95% CI will be used for all comparisons. R (version 3.6.1, The R Foundation) will be used for all statistical analysis [39].

### Missing Data

Each researcher is responsible for ensuring that any missing data are reported as missing in the study database. Procedures can sometimes be considered when using statistical methods that fail in the presence of any missing values, or in the case of multiple-predictor statistical models, all the data for an individual would be omitted because of a missing value in one of the predictors.

### Methods to Ensure the Validity and Quality of Data

Accurate and reliable data collection will be assured through verification and cross-checking of the electronic case report form (CRF). Discrepancies and queries will be generated accordingly in the CRF for web-based resolution by the researcher. In addition, the CRF data will be reviewed on an ongoing basis for scientific plausibility.

## Results

This study (ClinicalTrials.org trial# NCT04385797 [40]) was approved by the North-East Malaga Ethics Committee (1078-N-20) on April 30, 2020. Participants will provide their written consent before participating in the study. Substantial amendments that require review by the ethics committee will not be implemented until they are granted favorable opinion for the study. As of May 2021, a total of 153 participants have been enrolled and assessed during COVID-19 confinement, of whom 67 have been assessed at 6 months.

## Discussion

If our study hypothesis is accepted, these findings will demonstrate the negative impact of social isolation due to COVID-19 confinement on cognition, QoL, mood, and perceived stress, and technophilia in community-dwelling older PMCI/MDs, caregiver burden, and the access to and utilization of health and social care services, along with the cognitive, social, and entertainment-related use of ICTs during and after COVID-19 confinement.

Our findings will help assess the adoption of dementia technology by older people and their caregivers during the COVID-19 pandemic and may help alleviate the impact of present and future confinements in different aspects of their daily lives.

The preparedness and responses of governments to situations of the magnitude of COVID-19 will help determine related outcomes and consequences, which extend beyond the disease itself, with political, economic, cultural, health-related, and social impacts compromising the QoL of all. These responses, which include enforcing restrictive measures, lead to social isolation, which, in the context of an ecological framework, has an impact at the individual, relationship, community, and societal levels [4].

At an individual level, those with personal characteristics such as old age, life-course transitions, and health decline [10], particularly those with a diagnosis of dementia [3], are at higher risk. Abrupt and forced social isolation may be considered a disruptive event among older people and may predispose them to psychological distress [10]. Not being able to take part in daily activities, loss of usual routine, and nonattendance to memory workshops and day care services under closure may worsen cognition and functioning in this population. Daily activities such as going to grocery stores and pharmacies, may be carried out by caregivers to prevent risk of exposure, thus increasing caregiver burden.

At a relationship level, social distancing has compromised the reliability of social networks and the frequency of contact with others. Inadequate social support, when most needed, may aggravate depressive symptoms [12].

At a community level, interventions for the provision of health care and social support services are limited to prevent risk exposure, causing direct and indirect impacts on the prevention of ill health, promotion of health, and lifelong management and treatment of diseases.

At a societal level, social participation has been inhibited by policies that discourage social, economic, cultural, and physical activities. Regular participation in meaningful activities, including physical, intellectual, recreational, and social activities, serves beyond simple entertainment among PMCI/MDs, and discontinuing their participation in these activities may worsen cognition and regular functioning, thus increasing their dependence on instrumental activities of daily living [7].

Considering the extensive penetration of ICTs at home, home-based technological interventions have emerged for preventing health-related negative outcomes at all levels, including providing individual home-delivered cognitive stimulation, facilitating information sharing, and entertainment in daily life, fostering relations and social connectedness, enabling the delivery of routine health care and social support, preventing the risk of viral exposure, and offering home-based leisure activities. However, special attention must be paid to the use of ICTs among older people owing to the age-related digital divide and health-related conditions that compromise the use of ICTs (cognitive, visual, motor, etc). Fortunately, prior to this outbreak, Europe has, for several years, proactively invested in research and innovation programs in ICTs and their adaption among at-risk populations, promoting widespread, personalized, and accessible ICTs for all.

### Limitations

Although telephonic interviews would be the safest means to communicate with the dyads during the COVID-19 pandemic, there will be several drawbacks. The amount of information gathered and provided on a single telephone call is limited and researchers will have to balance the time spent on each call. Moreover, the assessment of cognition will be partial as the language section of the MMSE requires the assessment of visual and motor skills, which cannot be accomplished through a telephone call. Although evidence for telephone-based cognitive assessment recommends using the telephone interview for cognitive status instrument and its modified version [41,42], the study population undertook the MMSE thrice over a period of 1 year prior to this study, thus providing relevant data to compare their decline before confinement (baseline data) and afterwards. Furthermore, overloading PMCI/MDs with a long interview is not advisable as it may feel tedious and time-consuming. Finally, older people may have hearing impairments, which may make telephone assessments difficult. Caregiver assistance will be requested to complete the interviews.

The assessments in this study involve minimal risk to the patients. To ensure the standardization of interventions, researchers have received web-based training on the standard operating procedure, which includes a working plan, different aspects of the study, the protocol scheme to identify the appropriate measures, and the details to assess each variable safely among older adults. This will guarantee that the procedure can be replicated elsewhere.

## Abbreviations

CONNECTDEM: Cognitive Outcomes During COVID-19 confiNemeNt in Elderly and Their Caregivers Using Technologies for DEMentia
CRF: case report form
ICT: information and communications technology
MMSE: mini-mental state examination
PMCI/MD: people with mild cognitive impairment or mild dementia
QoL: quality of life
QoL-AD: Quality of Life-Alzheimer’s Disease
SMART4MD: support, monitoring, and reminder technology for mild dementia
TV-AssistDem: television-based assistive integrated service to support European adults living with mild dementia or mild cognitive impairment
